# Engineered Microvascular Model of the Blood–Brain–Tumor Barrier Reveals Endothelial Remodeling in Diffuse Midline Glioma

**DOI:** 10.1002/adhm.71474

**Published:** 2026-07-28

**Authors:** Kimberly R. Bennett, Yann S. Vanrobaeys, Tessy Teboh, Zakiya Smith, Anderson Scott, Cynthia Hajal, Paula T. Hammond, Joelle P. Straehla

**Affiliations:** ^1^ Harvard‐MIT Health Sciences & Technology Massachusetts Institute of Technology Cambridge Massachusetts USA; ^2^ Koch Institute for Integrative Cancer Research Massachusetts Institute of Technology Cambridge Massachusetts USA; ^3^ Department of Biochemistry Wellesley College Wellesley Massachusetts USA; ^4^ Department of Chemical Engineering Massachusetts Institute of Technology Cambridge Massachusetts USA; ^5^ Department of Mechanical Engineering Northeastern University Boston Massachusetts USA; ^6^ Ben Towne Center for Childhood Cancer & Blood Disorders Research Seattle Children's Research Institute Seattle Washington USA; ^7^ Department of Pediatrics University of Washington Seattle Washington USA

**Keywords:** blood–brain barrier, diffuse midline glioma, organ‐on‐a‐chip, pediatric neuro‐oncology, tumor microenvironment, tumor–vascular niche, vascular networks

## Abstract

Diffuse midline glioma (DMG) is a highly aggressive pediatric brain tumor that is difficult to treat because the blood–brain–tumor barrier (BBTB) prevents most drugs from reaching the tumor. A major obstacle for understanding this tumor‐vascular niche is the lack of a perfusable vascular model of DMG. To address this, we have established an in vitro model of the DMG‐BBTB, comprised of patient‐derived DMG tumor cells and primary human brain microvascular endothelial cells, astrocytes, and vascular pericytes, embedded in a fibrin matrix within a microfluidic chip. We report the development and characterization of self‐assembled microvascular networks, with consistent vasculature development across a range of tumor cell densities. Perfusion studies showed no significant alterations in vascular permeability when tumor cells were present. To elucidate transcriptomic changes, we employed scRNAseq on devices containing blood–brain barrier (BBB), DMG‐BBTB, or tumor necrosis factor alpha (TNFα)‐stimulated control. We observed significant differential gene expression between BBB and DMG‐BBTB, distinct from inflammatory signatures seen with TNFα perturbation. Our analysis indicated that DMG cells adopted a mesenchymal‐like phenotype, with further in silico studies predicting significant receptor‐ligand crosstalk between these cells and endothelium. In summary, we developed a high‐fidelity DMG‐BBTB model that recapitulates transcriptomic features of patient DMG microvasculature and features perfusable vasculature.

## Graphical abstract

We developed a patient‐derived, functional microfluidic model of the diffuse midline glioma (DMG) blood–brain–tumor barrier (BBTB) comprised of endothelial cells, astrocytes, pericytes, and tumor cells. The system forms perfusable microvasculature, reveals the BBTB retains vascular integrity, identifies DMG‐specific transcriptomic changes distinct from sterile inflammation, and predicts tumor–endothelial crosstalk, providing a high‐fidelity platform to study DMG vascular biology.

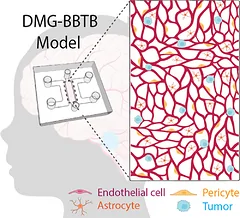

## 1 Introduction

Pediatric diffuse midline glioma (DMG) is an aggressive, fast‐growing, and infiltrative tumor [1]. Patients with DMG face dismal outcomes, with a 5‐year overall survival rate below 5% and most surviving less than 12 months [2]. The diffuse nature and location in vital midline brain structures present substantial surgical challenges. Consequently, radiotherapy remains the sole standard of care [3]. DMG is defined by both high‐grade histology and characteristic alterations in the H3 histone gene. Despite hundreds of clinical trials for new therapeutics, clinical outcomes have seen little improvement and no drug therapies have received frontline approval [4]. The failure of systemic drug therapies to extend survival has been widely attributed to the blood–brain barrier (BBB) [5]. Composed of endothelial cells, astrocytes, and pericytes, the BBB restricts drug transport into brain tissue, preventing therapeutic concentrations from reaching the tumor [6].

One major challenge in developing therapeutics for DMG lies at the blood–brain–tumor barrier (BBTB)—the intricate interface between existing brain vasculature and invading tumor cells [7]. In DMG, studies suggest that the vasculature often remains largely intact, maintaining its barrier function and preventing most drugs from entering the tumor microenvironment [8]. This finding directly challenges the long‐held assumption that all brain tumors exhibit compromised vasculature and underscores the critical need to specifically study the BBTB in DMG. Understanding the signaling and functionality of the BBTB is crucial to developing strategies that enhance drug penetration without harming healthy tissue.

Despite the critical importance of understanding the DMG‐BBTB for therapeutic development, a notable disconnect persists within the preclinical model pipeline, as many drugs that appear promising in vitro ultimately fail to demonstrate efficacy in the clinic [9]. Furthermore, existing in vitro models face limitations in specifically probing the BBTB in the context of DMG. While recent advances in preclinical modeling for DMG, such as microglia‐containing organoids, have emerged to evaluate the DMG tumor‐immune interface [10], the ability to explore vascular interactions is limited. Various co‐culture systems between DMG tumor cells with endothelial cells [11], as well as astrocytes and pericytes [12], have been reported; however, these systems lack the three‐dimensional (3D) vessel geometry characteristic of functional vasculature. This highlights the need for a platform that is more representative of human physiology to probe vascular biology and predict drug delivery.

To address this need, we developed a self‐assembling microvascular model of the human DMG‐BBTB that recapitulates the vascular networks and signaling pathways observed in DMG tumors [13]. Building on our prior work examining the BBTB in other glioma types [14], we engineered a DMG‐specific model. We demonstrate that the DMG‐BBTB model faithfully recapitulates the 3D structure and transcriptomic profile of human microvasculature. This robust system enables both quantitative solute perfusion studies and in‐depth investigation of key vascular‐tumor interactions. We establish the model's fidelity through orthogonal approaches, including direct comparison of vascular development in tumor (BBTB) versus non‐tumor (BBB) environments, and comprehensive single‐cell RNA sequencing benchmarked with established patient datasets. Our DMG‐BBTB model advances preclinical modeling of vascular‐tumor interactions for DMG, offering a human‐representative platform ideal for investigating the tumor microenvironment and developing novel therapeutics.

## 2 Results

### 2.1 A High‐Fidelity Model of the DMG Blood–Brain–Tumor Barrier

To recapitulate the DMG‐BBTB in vitro, we developed a microfluidic model featuring perfusable microvascular networks. This model incorporates a patient‐derived DMG tumor cell line, primary human brain microvascular endothelial cells (HBMEC), primary human brain vascular pericytes (HBVP), and primary human astrocytes (HA). The design expands on our previous work establishing an adult glioblastoma BBTB model around a tumor spheroid [14]. The pediatric DMG patient‐derived cell line BT245 was chosen for initial studies as a well‐characterized model from a thalamic DMG with H3.3K27M alteration, TP53 L194R mutation, and PDGFRa amplification. A second DMG model from a pontine source was also able to form perfusable vascular networks (BT869, H3.3K27M altered with ACVR1 mutation, Figure S1).

In this study, we engineered a DMG‐specific human microvascular model by co‐seeding tumor and vascular cell types in a fibrin gel matrix, which self‐assembled to form a 3D microvascular network alongside the diffusely spread tumor cells within the microfluidic device (Figure 1a). On day 3, an endothelial monolayer was added to flank both media channels and create a barrier between the media and central cell channels, as previously described [15]. Additionally, interstitial flow was established by adding media ports to one media channel to generate a hydrostatic pressure differential of 25 mm, with media being replenished daily. Microvascular networks fully formed after 7 days of culture in devices, with vessel lumens beginning to open after the addition of endothelial monolayer and hydrostatic pressure differential (Figure 1b,c). Endothelial cells and BT245 tumor cells were visualized using an expressed fluorescent reporter, as described in methods (Figure 1d). Pericytes and astrocytes were visualized after device fixation and staining for PDGFRb and GFAP, respectively (Figure 1d). The inclusion of tumor cells alongside developing microvasculature in this model enables the study of early tumor invasion and cellular influences on vasculature at the infiltrative margin.

**FIGURE 1:**
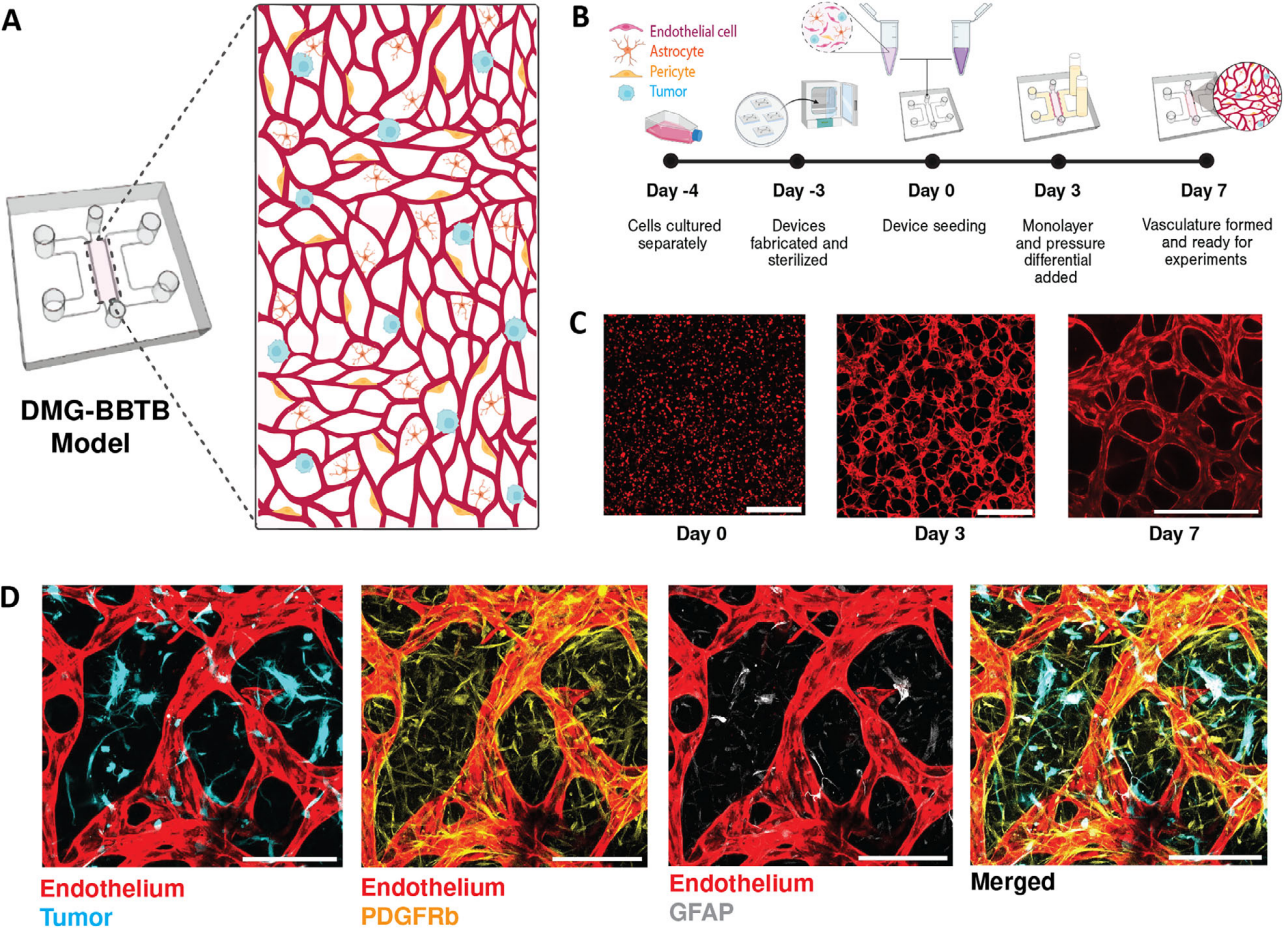
Self‐organizing microvascular model of the blood–brain–tumor barrier in the setting of diffuse midline glioma. (A) Schematic representation of the DMG‐BBTB model, containing patient‐derived tumor cells, primary human brain microvascular endothelial cells, astrocytes, and brain vascular pericytes grown in a fibrin gel matrix within a central microfluidic channel. (B) Timeline for creating DMG‐BBTB model, such that vasculature is fully formed on day 7 and ready for experimentation. (C) Endothelial vessel formation visualized using endothelial cells stably expressing fluorescent marker. Images are Z‐projections based on a stack of images taken on confocal microscope. Scale = 200 µm. (D) Visualizations of fully formed DMG‐BBTB model on day 7 with endothelial cells (red), BT245 tumor cells (cyan), pericytes stained for PDGFRb (magenta), and astrocytes stained for GFAP (yellow). Scale = 200 µm.

### 2.2 DMG Tumor Cells Do Not Substantially Alter Vascular Morphology or Integrity

Leveraging our model's ability to study tumor–vascular interactions, we first investigated a key aspect of DMG biology: vascular integrity. Contrary to the prevailing assumption that all vasculature is disrupted in high grade gliomas [16], patient and model mouse data allude to the presence of a “super‐BBB”, where vasculature is more restrictive in the pons, thereby limiting delivery more significantly compared to vessels in the cortex [17]. Similar observations in genetically engineered mouse models of DMG confirm a largely intact vasculature in the brainstem [8]. To assess our model's fidelity in replicating vascular integrity, we directly compared microvasculature development in BBB devices without tumor cells to BBTB devices containing either low (5000 cells/device) or high (25 000 cells/device) tumor density. Specifically, we examined changes in vessel diameter, branching, and permeability between devices with and without tumor cells.

We observed no appreciable morphological differences between BBB devices and BBTB devices of low or high tumor density (Figure 2a). To quantify this, we measured vessel diameters in both the transverse and the lateral dimensions to capture the 3D geometry of the microvasculature [18]. We found no significant change in vessel diameter across device conditions after two‐way ANOVA statistical analysis (Figure 2b). We then quantified vessel branching, defined as the number of vessel branch points per image area, across devices using skeletonized masks of vessels following an established protocol [18]. Skeletons were then used to calculate the number of branch points within the image as well as the lengths between each point. Notably, all microvascular networks across conditions featured a large number of vascular branching (over 100 branches per image area), differentiating this model of the DMG‐BBTB from other existing microfluidic‐based models of DMG. However, vessel branching was not significantly altered between BBB and BBTB conditions, measured via one‐way ANOVA (Figure 2c). Similarly, branch lengths, or the length of vessels between branch points, remained unaltered across devices conditions (Figure 2d). These findings suggest that microvascular morphology is maintained in the presence of low‐ and high‐density tumor cells. We also tested a model of an ultra‐high tumor density (50 000 tumor cells/device) and found that this density prevented the reliable formation of perfusable vessels (Figure S2). Because these devices could not be reliably perfused, they were not suitable for our downstream assays. Therefore, we moved forward with evaluating the permeability of BBTB devices with initial tumor cell densities of 5000 and 25 000 tumor cells per device.

**FIGURE 2:**
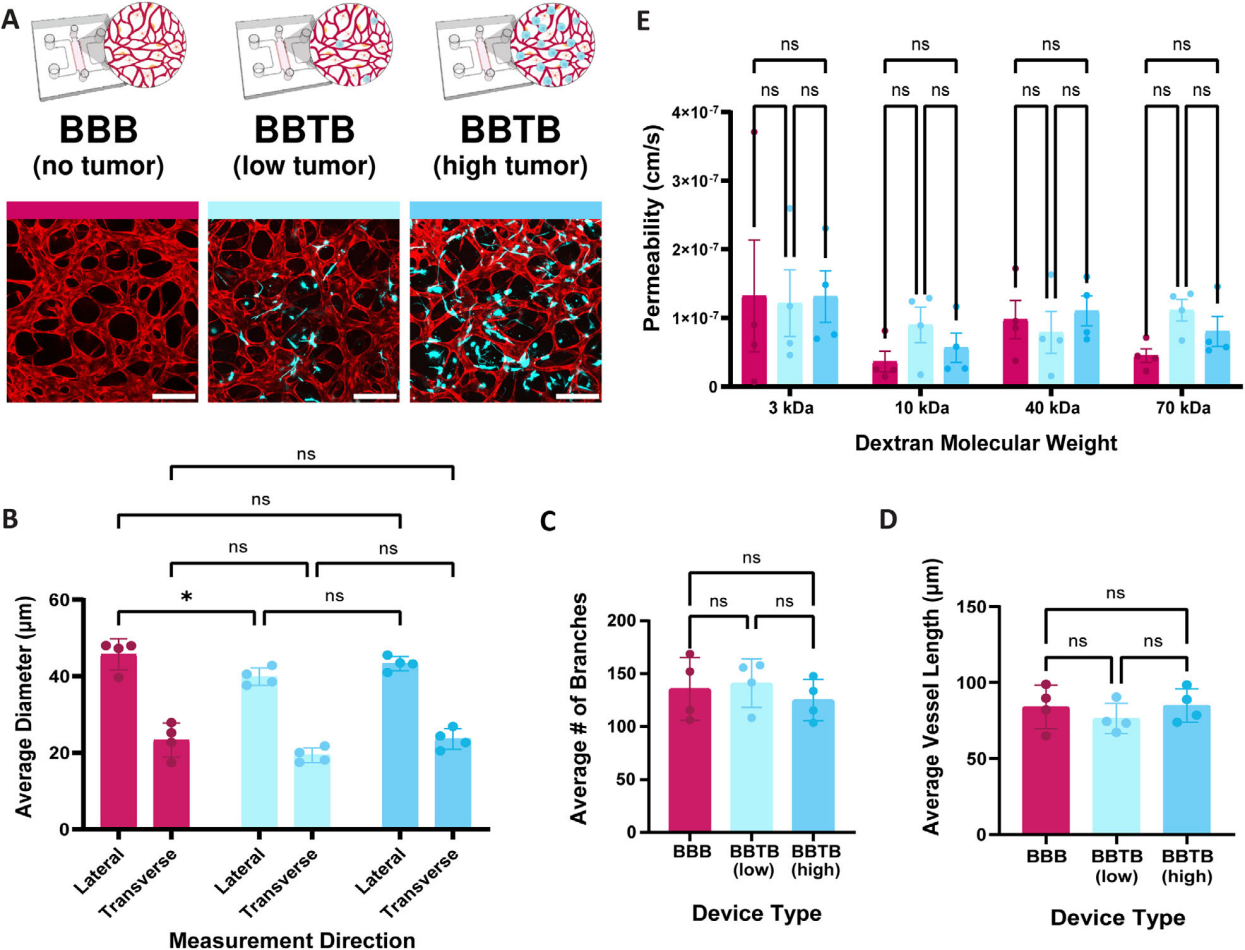
Microvasculature morphology and permeability remain unaltered in the presence of diffuse midline glioma cells. (A) Representative overview of model device conditions: No tumor control (BBB only), 5000 tumor cells (BBTB‐low), and 25 000 tumor cells (BBTB‐high). Corresponding confocal images of vasculature (red) and tumor (cyan) amongst conditions. Scale = 100 µm. (B) Average vessel diameters, measured in lateral and transverse dimensions, for each condition. Values reported are average values from three ROIs per device per *n* = 1, *n* = 4 devices per condition. Center values depict average, with error bars denoting standard deviation. Significance measured through ordinary two‐way ANOVA with Šídák's multiple comparisons test, * = *p* < 0.05, ** = *p* < 0.01, ns = not significant. (C) Average number of vessel branches per device type, per image area. Values reported are average values from three ROIs per device per *n* = 1, *n* = 4 devices per condition. Center values depict average, with error bars denoting standard deviation. Significance measured through ordinary one‐way ANOVA with Tukey's multiple comparisons test, * = *p* < 0.05, ** = *p* < 0.01, ns = not significant. (D) Average vessel branch length per device type, defined as length between two branch points. Values reported are average values from three ROIs per device per *n* = 1, *n* = 4 devices per condition. Center values depict average, with error bars denoting standard deviation. Significance measured through ordinary one‐way ANOVA with Tukey's multiple comparisons test, * = *p* < 0.05, ** = *p* < 0.01, ns = not significant. (E) Average permeability (cm s^−1^) of 3, 10, 40, and 70 kDa dextran across device types. Values reported are average values from three ROIs per device per *n* = 1, *n* = 4 devices per condition. Center values depict average, with error bars denoting standard error. Significance measured through ordinary two‐way ANOVA with Šídák's multiple comparisons test, * = *p* < 0.05, ** = *p* < 0.01, ns = not significant.

To examine the functional integrity of microvasculature in the presence of DMG, we assessed paracellular permeability by perfusing fluorescently labeled dextrans and measuring changes in extravascular intensity over time. We perfused dextrans of 3, 10, 40, or 70 kDa molecular weights through BBB, BBTB‐low, and BBTB‐high devices (*n* = 4 each), and tracked the solute via confocal imaging over 12 min. Permeability values were calculated as previously described [18], where the collected time‐lapse intensity data allows us to determine the permeability coefficient as detailed in the methods [19]. Permeability values in the BBB model were consistent with literature benchmark reported in previous microvascular models [20] and in mice perfusion studies [21]. Comparing BBB control to DMG‐BBTB devices via two‐way ANOVA statistical analysis, there was no statistically significant difference in permeability across conditions to 3, 10, 40, or 70 kDa dextran (Figure 2e). Additionally, permeability values were similar across all conditions tested and averaged less than 1 × 10^−6^ cm s^−1^, consistent with a highly restrictive biologic barrier [22]. Taken together, morphologic and functional data from the DMG‐BBTB model demonstrate that the microvasculature remains intact and functionally unaltered in the presence of invading tumor cells at both low and high densities.

### 2.3 High Resolution scRNAseq Defines the DMG‐BBTB Vascular Niche

To further probe the biologic fidelity of our BBTB model, we employed single‐cell RNA sequencing (scRNAseq) to gain high resolution insights into cellular subtypes and changes due to the presence of tumor cells or an inflammatory challenge. Three variations of the microvascular model were generated for this study: BBB cells only, BBTB devices seeded with 25 000 tumor cells, BBB cells challenged with tumor necrosis factor‐alpha (TNFα) to induce sterile inflammation. As TNFα is a marker implicated in early vascular response to radiation [23], where it is shown to be upregulated following radiation exposure [24], it was chosen as a pathological control to distinguish tumor‐specific vascular remodeling from general inflammatory barrier disruption or radiation‐induced damage. After 7 days of self‐assembly, microvascular network formation was verified by confocal microscopy before samples were prepared for sequencing. Fibrin gels containing cells were extracted from the central microfluidic channel and enzymatically digested to obtain single cell suspension of cells (Figure 3a). Six total devices were processed and analyzed, resulting in 87 889 evaluable cells.

**FIGURE 3:**
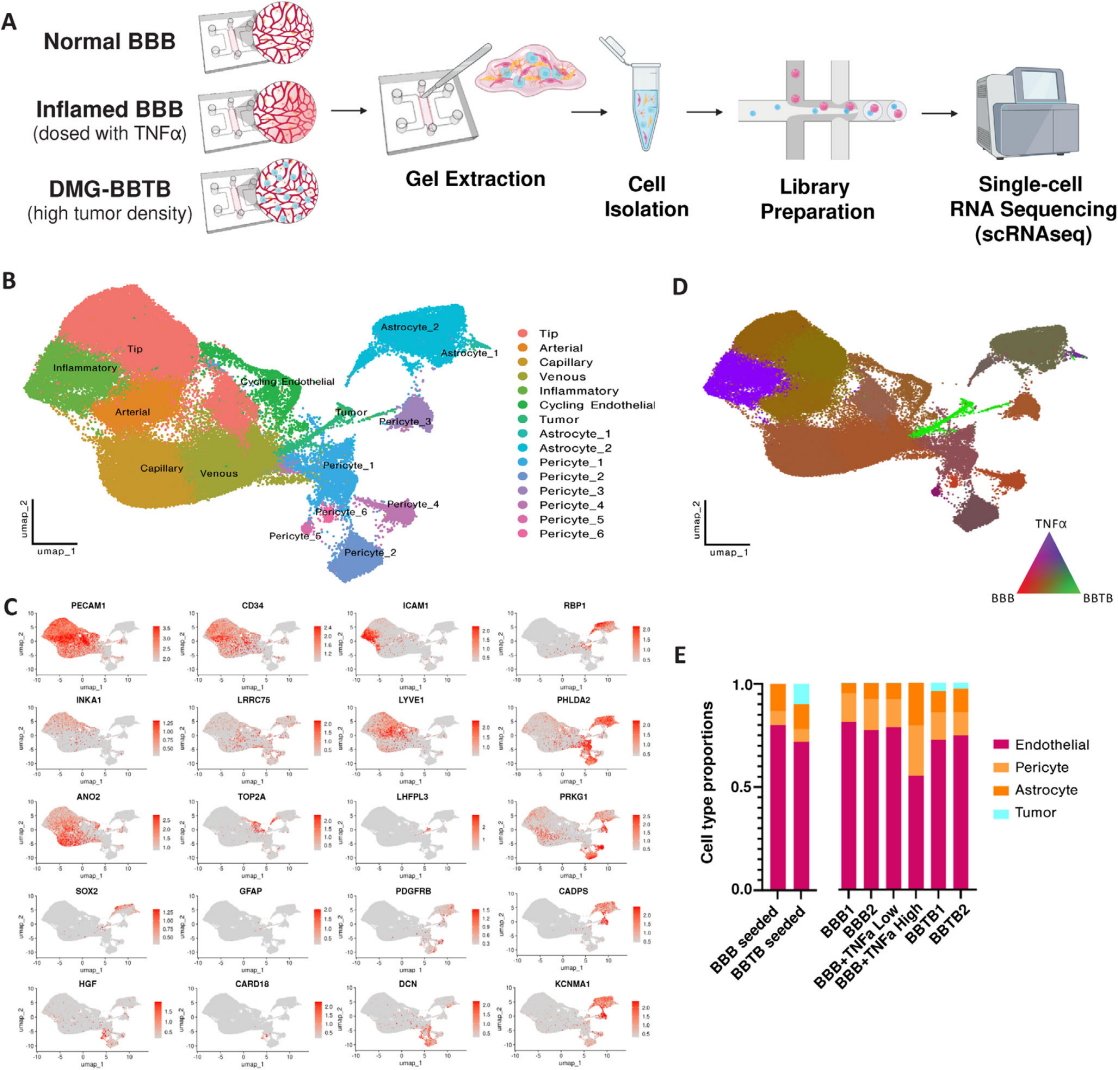
Microvascular models recapitulate human microvascular endothelial and stromal subtypes. (A) Schematic workflow of single‐cell RNA sequencing of devices. BBB devices (*n* = 2), inflamed BBB devices dosed with TNFα (*n* = 2), and BBTB devices with high tumor density (*n* = 2) were sequenced. (B) Detailed cell annotations for all cell populations. (C) Corresponding feature plots of top markers used to define subtyping. Cells were profiled against published human vascular development datasets to establish human relevance. (D) Contribution of cells by sample source is depicted, with color corresponding to sample type of BBB, BBTB, or BBB+TNFα. (E) Proportions of cell types within sequenced samples compared to seeded densities.

Unsupervised clustering conducted using the Louvain algorithm implemented in Seurat's FindClusters() function led to the identification of 17 total clusters, including 6 endothelial (EC), 8 stromal, and 1 tumor cluster (Figure 3b and Figure S3). Major cell populations were confirmed using canonical markers: ECs (CD34+ and PECAM1+), stromal cells (PDGFRb+), and tumor cells (LHFPL3+) (Figure 3c). The six subclusters of endothelial cells were identified based on expression of known functional markers representing tip‐like (INKA1+), capillary‐like (ANO2+), cycling (TOP2A+), venous‐like (EphB4+), arterial‐like (LYVE1+), and inflamed (ICAM1+) ECs through direct comparison with prenatal human brain vascular development datasets [25]. Condition‐specific cell clusters were observed, with a unique EC subpopulation appearing solely in the BBB+TNFα samples and, as expected, the tumor cell cluster was unique to BBTB samples (Figure 3d). Overall, cell type proportions were maintained across conditions and consistent with seeding conditions (Figure 3e). Endothelial cells were the most abundant cell type, yet there was a notable decrease in ECs and increase in pericytes in devices treated with high‐dose TNFα, consistent with the documented role of pericytes in inflammatory response [26].

Sterile inflammation was induced using TNFα as a control to demonstrate that the microvasculature in the model can respond to inflammatory perturbation [27], and distinguish tumor‐specific vascular remodeling from general inflammatory barrier disruption or radiation‐induced damage. BBB devices were dosed with TNFα (TNFα Low: 1 ng mL^−1^ or TNFα High: 2.5 ng mL^−1^) for 1 h prior to imaging and sequencing. TNFα High devices exhibited significant vascular disruption with decreased dextran perfusion, while TNFα Low were morphologically similar to untreated BBB devices (Figure 4a). This functional change in perfusion was consistent with a dose‐dependent reduction in endothelial cell number observed by single‐cell analysis in TNFα High devices (Figure 4b). Despite the absence of vascular disruption in the TNFα Low condition, both TNFα samples exhibited a conserved inflammatory endothelial signature (Figure 4b). This demonstrates that transcriptomic changes can precede or occur independent of overt vascular disruption in this model system. Specifically, endothelial cells in both TNFα conditions featured increased expression of inflammatory genes VCAM‐1, ICAM1, CXCL8, and IL6 (Figure 4c). Further, canonical BBB integrity markers, including PECAM1, TJP1, and OCLN were downregulated in a dose‐dependent manner (Figure 4c). This cytokine challenge established that the base BBB model is responsive to inflammatory stimuli.

**FIGURE 4:**
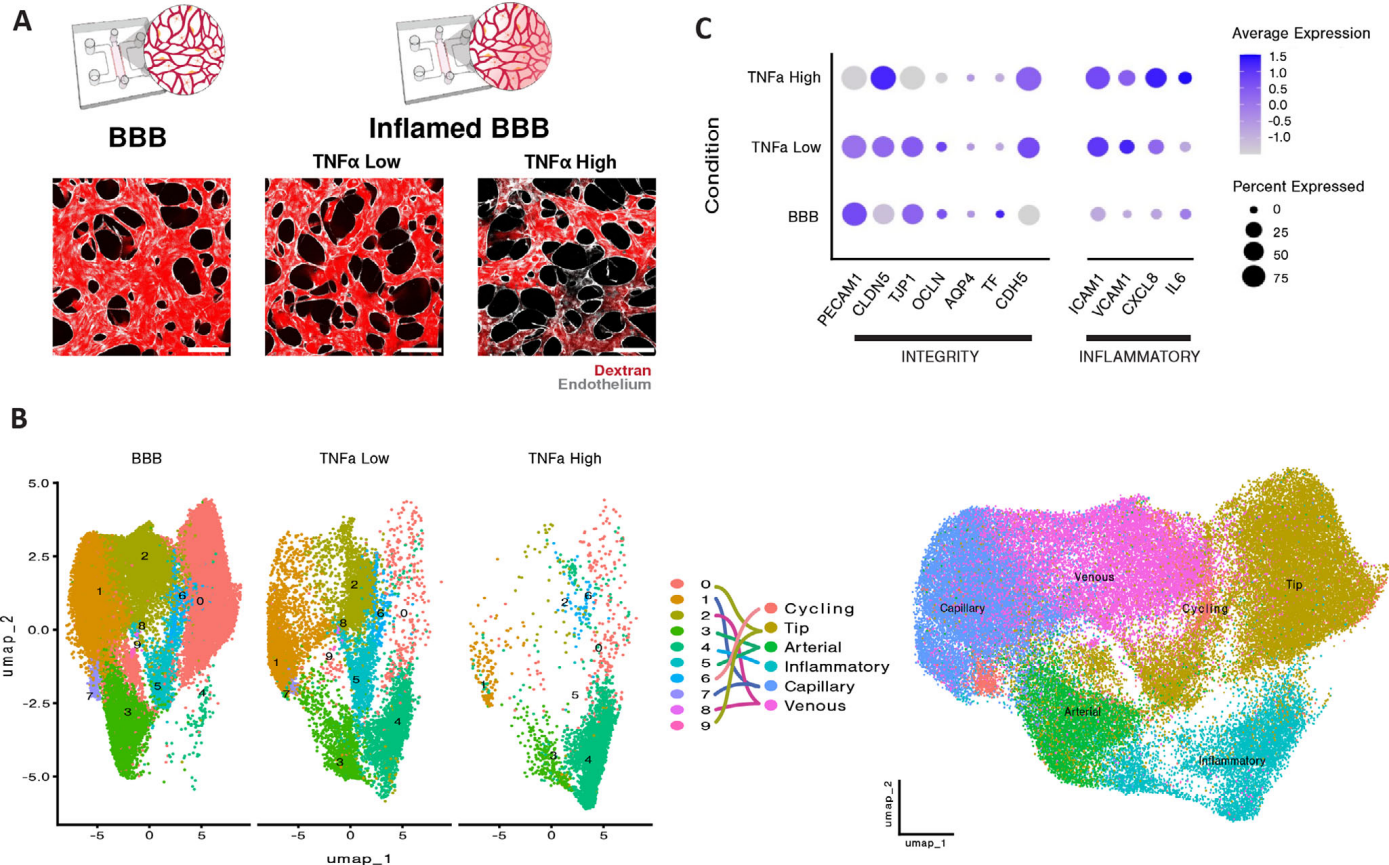
Perturbable microvasculature exhibits a strong inflammatory response to TNFα cytokine dosage. (A) Overview of cohort examined. BBB devices (*n* = 2) and Inflamed BBB devices, at a low dose (1 ng mL^−1^, *n* = 1) and high dose (2.5 ng mL^−1^, *n* = 1) of TNFα for 1 h prior to collection, were evaluated. Low and High inflammatory conditions were used to contrast perturbations in vascular biology when vessels were not visually disrupted to a high dose where vascular perfusion began to be compromised. Confocal images of devices to be sequenced were taken, with 70 kDa dextran perfused to confirm vascular morphology and perfusability. Scale = 100 µm. (B) Corresponding scRNAseq clusters of endothelial cells within each respective device condition that were compiled for analysis. (C) Expression of BBB integrity and inflammatory markers in endothelial cells across conditions.

### 2.4 The DMG‐BBTB Exhibits a Distinct Transcriptomic Profile

We next examined the endothelial cell profiles across BBB and BBTB samples. In direct contrast to the unique inflammatory endothelial phenotype induced by TNFα, the presence of DMG cells did not induce an inflammatory expression profile in BBTB samples (Figure 5a). Inflammatory marker expression (VCAM1, ICAM1, CXCL8, and IL6) remained low and largely unaltered between BBB and BBTB endothelial cells (Figure 5b). Instead, we observed an increase in BBB integrity markers (CLDN5, TJP1, AQP4, and CDH5) in BBTB conditions compared to control BBB, suggesting enhanced endothelial barrier regulation in the presence of tumors [28] and consistent with our permeability data demonstrating intact BBB vasculature (Figure 2e). Additional BBB integrity markers PECAM1, TF, and OCLN were conserved between conditions. Further analysis of endothelial transporters, including ATP‐binding cassette (ABC) transporters (ABCB1/P‐gp, ABCG2/BCRP, ABCC1/MRP1, ABCC2/MRP2, ABCA1, ABCG4, and ABCA7) [29], solute carrier (SLC) transporters (SLC2a1/GLUT1, SLCO1a2/OATP1a2, SLC22a8/OAT3, SLC7a5/LAT1, and SLC22a3/OCT30) [30], and receptor‐mediated transcytosis (RMT) transporters (LRP1, LRP2, LRP8, AGER, INSR, LDLR, IGF1R, LEPR, and SLC18A2/VMAT2) [30] between BBB and BBTB conditions were evaluated (Figures S4, S5, and S6). These findings demonstrate that the DMG BBTB retains intact tight junction and structural integrity, features an increase in SLC transporter expression, moderate conservation of RMT transporters, yet mixed regulation of ABC transporters, with ABCG2/BCRP and ABCC2/MRP2 being slightly increased in BBTB capillary‐like ECs while other ABC transporters were conserved or slightly decreased in BBTB endothelial cells.

**FIGURE 5:**
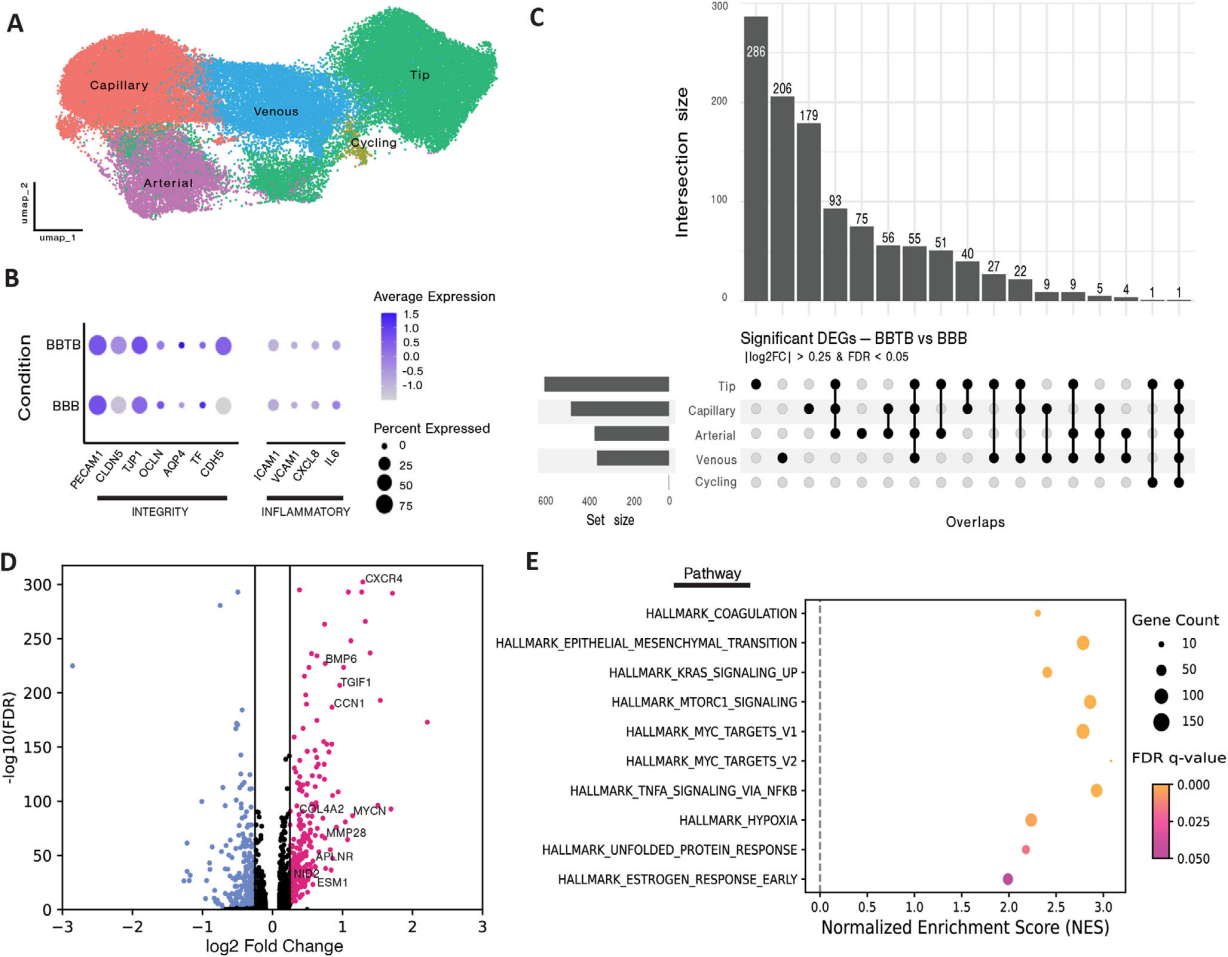
DMG tumor cells impart biological changes in microvasculature revealed by scRNAseq analysis. (A) Overview of cohort examined, where all endothelial cells present in BBB and BBTB samples were probed for analysis. Endothelial subtyping as previously described were used to annotate cell clusters. (B) Expression of BBB integrity markers across conditions, contrasting expression of BBB integrity markers to inflammatory markers to examine biological changes in endothelial cells across conditions. (C) Upset plot of endothelial cell subtype differential gene expression (DEG) patterns. Interaction sizes refer to the number of unique DEGs corresponding to the group, and set size refers to the total number of DEGs per cell type. Overlap bubbles refer to cell type groupings that express the corresponding DEGs. DEGs were calculated using a two‐sided Wilcoxon rank‐sum test and minimum cell percentage cutoff of ≥10% of cells. (D) Differential expression of genes in tip‐like endothelial cells (ECs) between BBB and BBTB conditions. Genes with an absolute fold change > 0.25 and an FDR‐adjusted *q*‐value < 0.05 were examined (E) Hallmark pathway enrichment of DEGs in tip‐like ECs between BBB and BBTB conditions evaluated via GSEA.

While canonical BBB integrity markers and transporters showed minimal differences, an unbiased analysis of endothelial cell expression between BBB and BBTB conditions revealed numerous differentially expressed genes (DEGs) across subtypes, with tip‐like ECs having the highest number of DEGs globally and had the most uniquely DEGs not observed in other subtypes (Figure 5c). Specifically, there were 286 unique DEGs in tip‐like ECs meeting statistical significance, defined by absolute fold change > 0.25 and false discovery rate (FDR) adjusted *q*‐value < 0.05. As such, we focused on examining the DEGs within the tip‐like EC subtype (full results of DEGs in all cell populations in Table S1). Genes with significant differential expression in BBTB tip‐like cells versus BBB (fold change > 0.25, *q*‐value < 0.05) were highlighted (Figure 5d). To determine the functional consequences of DMG‐induced transcriptomic changes in endothelial cells, gene set enrichment analysis (GSEA) was conducted. Examination of hallmark pathways enriched in the tip‐like EC dataset (FDR *q*‐value < 0.05) revealed 10 enriched pathways (Figure 5e, full results of GSEA in all cell populations in Table S2). Several of the enriched pathways converge on themes of cellular growth, survival, and aggressive tumor biology, including targets of the MYC transcription factor, epithelial‐mesenchymal transition (EMT), mechanistic target of rapamycin (MTOR) signaling, and TNFα signaling via nuclear factor kappa B (NFκB). The co‐enrichment of MTOR signaling and pathways related to MYC and EMT suggests a shift in endothelial cell phenotype towards proliferation and plasticity. This finding is notable because, although the DMG patient model used here lacks MYC overexpression or co‐occurring MTOR pathway alterations, DMG cells in a differentiated state have been shown to upregulate genes associated with MYC and MTOR signaling [31]. Further, the enrichment of NFκB pathway indicates presence of a pro‐survival, stress‐response signature despite the lack of overt inflammation. The identification of an EMT signature, a marker of endothelial plasticity, is particularly relevant as this process has been identified in other aggressive brain tumors as a driver of aberrant vascularization [32] and therapy resistance [33].

### 2.5 Mesenchymal‐Like Tumor Cells Drive Signaling Crosstalk

The EMT signature identified in endothelial cells suggests that DMG tumor cells may be driving vascular plasticity in our model. To test whether tumor cells within the DMG‐BBTB model themselves are in a mesenchymal‐like state, we evaluated the tumor cell cluster using published cellular metaprograms derived from a cohort of 43 pediatric patient tumors (Figure 6a) [34]. DMG tumors are known to exhibit a hierarchy of cell states, including oligodendroglial precursor cell (OPC)‐like, astrocyte (AC)‐like, mesenchymal (MES)‐like, or oligodendrocyte‐like (OC)‐like cell states [34]. While the BT245 tumor cells retained their OPC‐like state when grown as a neurosphere line in monoculture (Figure 6b), tumor cells grown within the BBTB devices shifted toward the MES‐like state seen in patient tumor samples (Figure 6b). Direct comparison of representative metaprogram marker expression showed that tumor cells from the DMG‐BBTB devices highly expressed MES‐like markers such as secreted phosphoprotein 1 (SPP1), tissue inhibitor of metalloproteinase 1 (TIMP1), DNA‐damage inducible transcript 3 (DDIT3), and growth associated protein 43 (GAP43), compared to monoculture (Figure 6c).

**FIGURE 6:**
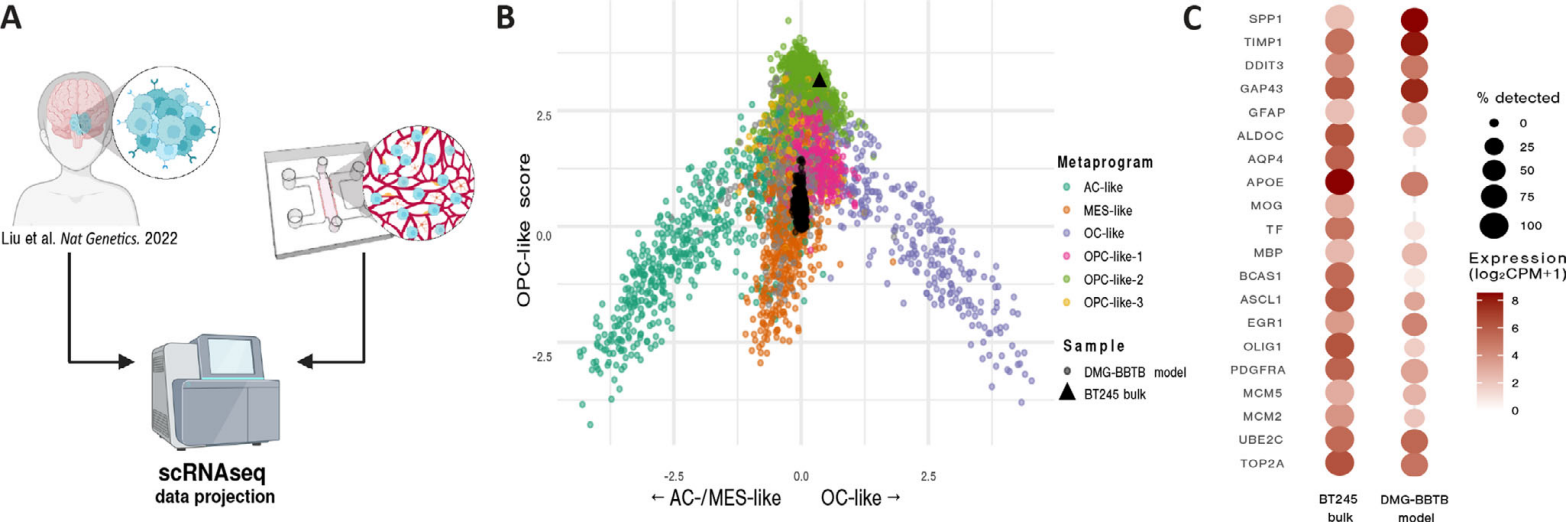
DMG‐BBTB model recapitulates MES‐like tumor cell states seen in patient H3K27‐altered gliomas. (A) Overview of cohort examined. scRNAseq data of tumor cells within BBTB devices were projected against an established dataset from Liu et al. containing a cohort of H3K27M pediatric DMG tumor samples. (B) Ranking of tumor cell metaprogram on a scale of OPC‐like, AC‐like, MES‐like, and OC‐like. Hierarchy of metaprograms are shown in contrast to bulk RNAseq of the BT245 cell line (black triangle) and scRNAseq data of tumors within the DMG‐BBTB model (black dots). Patient data pulled from Liu et al. 2022, with their original annotations of AC‐like (teal), MES‐like (orange), OC‐like (purple), OPC‐like cells (pink, green, and yellow) plotted for comparison. (C) Expression of metaprogram markers in the tumor cells within the DMG‐BBTB model compared to BT245 monoculture.

To elucidate mechanisms of vascular influence by tumor cells, we performed in silico predictions of ligand‐receptor interactions of tumor cells and vasculature using the LIANA (LIgand–receptor ANalysis) platform [35]. In this analysis, multiple ligand–receptor interaction resources are aggregated to compute ranked predicted interactions between DMG tumor cells and endothelial cell subtypes (Figure 7a). Analysis between all cell populations, including astrocytes and pericytes, was also computed (Table S3). Consistent with previous results showing that tip cells were highly differentially regulated (Figure 5c), the tip cells featured the most ranked predicted interactions with tumor cells (Figure 7a).

**FIGURE 7:**
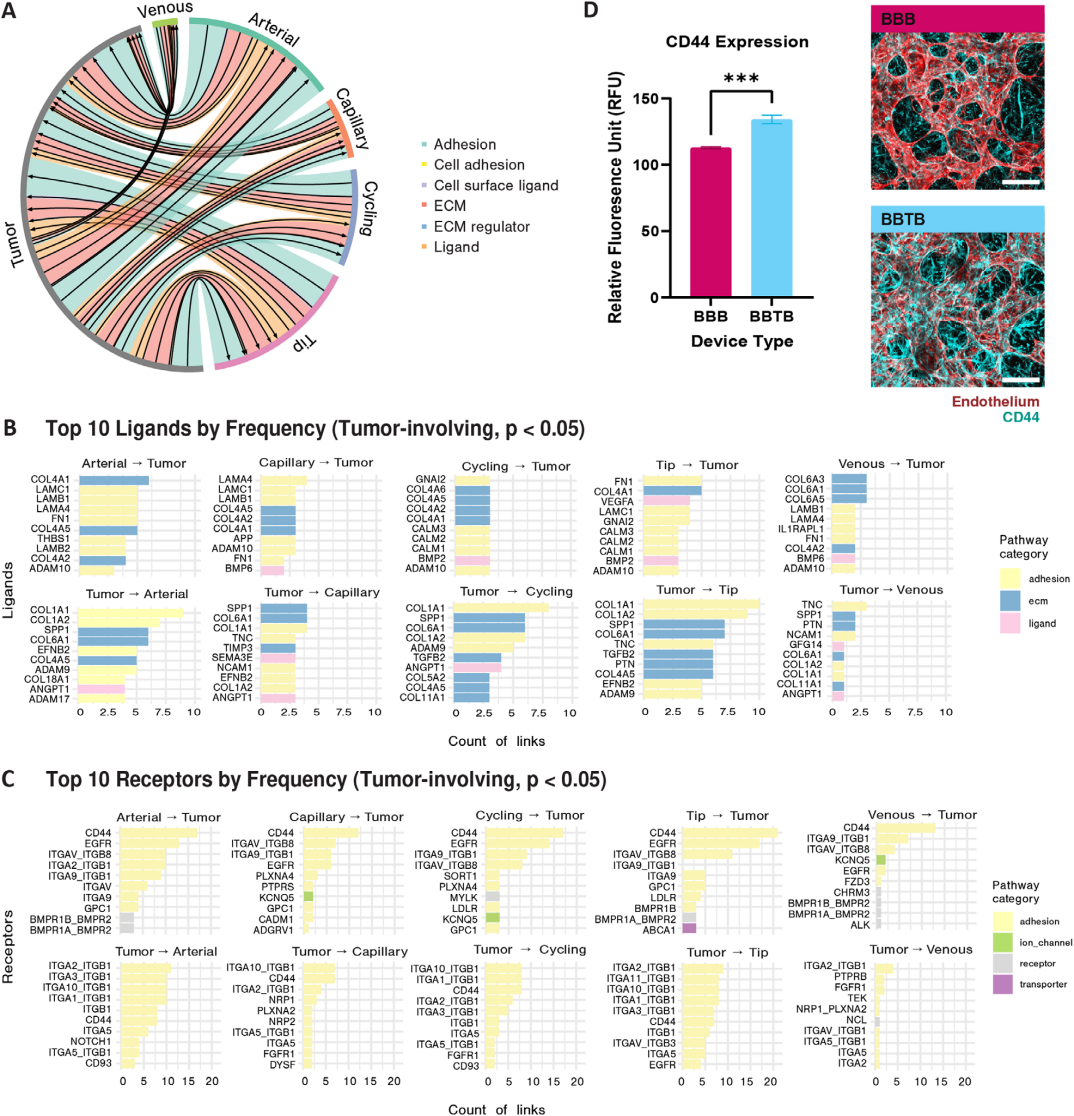
Ligand–receptor interactions of the DMG‐BBTB recapitulate patient characteristics, informing therapeutic strategies. (A) Network map of ligand–receptor interactions between tumor and endothelial cell subtypes. (B) Top 10 most frequent ligands on endothelial cells to tumor receptors and tumor cells to endothelial and stromal receptors. Color coding denotes pathway category these ligands correspond to. (C) Top 10 most frequent receptors on endothelial cells interacting to tumor ligands and tumor cells to endothelial ligands. Color coding denotes pathway category these receptors correspond to, grey referring to uncategorized. (D) Representative image of immunofluorescent staining of CD44 (cyan) and vasculature (red) in BBB vs BBTB devices. Image depicts a collapsed z‐stack of one device replicate ROI. Scale = 100 um (E) Quantification of relative fluorescence intensity of CD44 staining in BBB versus BBTB devices. Center values depict average, with error bars denoting standard deviation. Significance measured through *t*‐test, *** = *p* < 0.001.

We then ranked the top 10 most significant unique ligands and receptors between each endothelial subtype and DMG tumor cells to understand interaction frequency. For endothelial ligands targeting tumor receptors, the top hits primarily categorized into cell adhesion and extracellular matrix pathways (Figure 7b). Specifically, endothelial cell ligands corresponding to basement membrane proteins laminin (LAM) and type IV collagen (COL4) were the most abundant across endothelial cell subtypes. Conversely, highly abundant tumor ligands included collagen (COL1) and osteopontin (SPP1). For tumor receptors, cluster of differentiation 44 (CD44) interactions consistently ranked most frequent across all endothelial subtypes, alongside receptors for the aforementioned ligands such as integrins (ITGA/B subunits), and bone morphogenic protein (BMP) receptors 1 and 2 (Figure 7c).

To validate the key ligand‐receptor predictions, we performed immunofluorescence staining for CD44 on BBB and BBTB devices. Since SPP1 binding to CD44 has been utilized as a prognostic marker in other gliomas indicating invasive MES‐like cell state [36], we hypothesized that CD44 would be differentially expressed in devices. Quantified over *n* = 3, DMG‐BBTB devices stained for CD44 featured a significant increase in relative fluorescence intensity compared to the BBB control (*p*‐value < 0.001) (Figure 7d).

## 3 Discussion

In this study, we present a high‐fidelity in vitro model of the blood‐brain‐tumor barrier (BBTB) of diffuse midline glioma (DMG) that features perfusable human microvasculature grown with patient‐derived tumor cells. The DMG‐BBTB model, comprised of a co‐culture of DMG cells with primary human BBB cells embedded in a fibrin matrix within a microfluidic chip, provides a model that supports on‐chip cell differentiation and is compatible with multiple functional and biological assays. A major challenge in in vitro BBB modeling is that isolated brain endothelial cells lose their specialized properties, often resulting in dedifferentiation marked by a gene expression profile resembling epithelial cells [37]. In contrast, our single‐cell RNA sequencing data show that the vasculature not only avoids this dedifferentiation, but also faithfully maps to human developmental datasets and subcategorizes into multiple specialized EC subtypes (e.g., capillary, tip, venous). This high‐fidelity recapitulation of the vascular barrier enables functional probing of the DMG‐BBTB interface under physiologically relevant flow conditions.

We examined the tumor–vascular interface of pediatric DMG by characterizing the morphological, functional, and biological changes in the DMG‐BBTB model compared to BBB control. Morphologically, vasculature development in the presence of DMG was unperturbed, and functionally, vessel permeability remained largely unaltered between BBB and BBTB conditions. This suggests our model faithfully captures early, restrictive stages of tumor development, supporting the observed increase in BBB integrity markers (CLDN5, TJP1, etc.) in the BBTB condition. While vascular formulation was disrupted at ultrahigh tumor concentrations, potentially due to resource competition within the microfluidic architecture, this finding may be clinically relevant, as the dense core of pontine DMG has been shown to have reduced vascular density at late stages of disease [38].

Our transcriptomic analyses revealed that DMG cells do not impart the same acute inflammatory change upon endothelium as TNFα perturbation, demonstrating the model's utility as a perturbable platform capable of probing both extrinsic and cell‐based challenges. This contrast suggests a more complex, non‐inflammatory effect of DMG on vasculature. Instead of an inflammatory signature, GSEA showed significant enrichment of pathways related to cellular growth and plasticity, including MTOR signaling, MYC targets, and the EMT pathway. The enrichment of these growth‐factor and plasticity associated pathways confirms that while the BBTB remains restrictive in the presence of DMG, the endothelium is undergoing transcriptional remodeling in response to the tumor microenvironment.

Our data suggests that the strong EMT signature observed in endothelial cells was imparted by DMG cells with a MES‐like phenotype. This demonstration of on‐chip cellular differentiation to a clinically aggressive state is highly relevant, as this metaprogram was associated with increased vascularization and co‐localization with vascular cells in spatial transcriptomic analyses of DMG patient samples [34]. Furthermore, the MES‐like phenotype of endothelial cells has been associated with tumor progression [39] and resistance to therapy [40] in other high‐grade glial tumors, underscoring the need for preclinical models that can faithfully reproduce this state.

To delineate the mechanism of MES‐like tumor cell influence on vasculature in our model, in silico predictions and subsequent validation pinpointed key interactions. Specifically, CD44 and its ligand SPP1 consistently ranked as the top ligand‐receptor pair between tumor cells and endothelial cells, particularly the highly regulated tip cells. The importance of this ligand‐receptor axis was further supported by a significant upregulation of CD44 at the protein level in BBTB devices. Other consistent ligand‐receptor pairs including integrins and BMP receptors highlight additional targetable mechanisms by which DMG cells may interact with and remodel the microvasculature.

While we recapitulated important aspects of patient biology, including the development of MES‐like tumor cells and resulting vascular plasticity, our high‐resolution analysis also uncovered potential new targets that have not been previously associated with DMG. For example, genes such as cysteine‐rich angiogenic inducer 61 (CCN1, also known as CYR61) [41] and endothelial‐secreted endocan (ESM1) [42] have been associated with angiogenesis in adult gliomas, while matrix metallopeptidase 28 (MMP28) has been associated with migration [43] in glioblastoma (GBM). Additionally, TGFB‐induced factor homeobox 1 (TGIF1) [44] and nindogen‐2 (NID2) [45] have been associated with poorer prognosis in GBM, and yet these markers have not been implicated for DMG. CD146 (also known as MCAM), which was differentially expressed exclusively in tip‐like cells, has been associated with aggressiveness in adult GBM [46], and its upregulation on microvasculature has been correlated to tumor grading in adult‐type diffuse gliomas [47], yet has not been reported for pediatric DMG. Similarly, apelin receptor (APLNR) has been associated with angiogenesis‐dependent GBM growth [48], and apelin targeting has been under investigation to impair glioma progression [49]. In contrast, chemokine receptor type 4 (CXCR4) has been previously proposed as a target in a subset of pediatric DMG patients [50] and was significantly upregulated on DMG vasculature in our model. Consistent with our in‐silico findings, bone morphogenetic protein (BMP6) was upregulated in DMG vasculature, and the BMP family has been previously indicated in pediatric DMG cohorts [51]. Further comparison to GBM endothelial signatures from human datasets [52] demonstrated the enrichment of COL4A1 in both the GBM and DMG BBTB, yet DMG endothelial cells lacked changes in PLVAP expression previously reported in GBM [53] (Figure S7). This suggests that while there are some similarities, BBTB characteristics vary across glioma subtypes and underscores the necessity for DMG‐specific investigations. This identification of both new and established signaling nodes, and uniqueness of the DMG‐BBTB, highlights the potential of our functional model to probe the vascular–tumor interface with high resolution and accelerate the discovery of new targetable strategies for DMG.

Despite its high fidelity, our model has several limitations. We acknowledge that while this is a highly complex, 4‐cell type system with robust vasculature, it currently lacks the tumor microenvironment's innate immune cell representation. Given the critical role of myeloid cells, such as microglia and tumor‐associated macrophages, in DMG progression and therapeutic response [34], incorporating these elements is an important goal for future iterations of this model. Incorporation of peripheral immune components will enable the investigation of neuroimmune‐vascular interactions in the DMG BBTB and screening of immunotherapies using the platform. We also acknowledge that while we have extensively characterized one patient‐derived DMG model (BT245), future work must explore tumor region and interpatient heterogeneity. There is sufficient literature to suggest that DMG tumors in the pons and thalamus differ clinically [54] and biologically [55], so exploring these differences within the DMG‐BBTB model is an important future investigation. Beneficially, the DMG‐BBTB platform is highly versatile, as the core principles of this platform permit for the investigation of other tumor cell lines, indicated by our preliminary feasibility work characterizing the vascular changes driven by the patient‐derived pontine BT869 DMG line (Data S1). Another limitation to this model is the use of primary endothelial cells derived from the cerebral cortex. As the availability of primary brainstem cell lines improves, future iterations of this model can explore the regional differences in tumor–vascular interactions. Its robust recapitulation of tumor‐vascular interactions similarly permits investigation of other rare brain tumor types by swapping the DMG cells for other relevant tumor lines, as indicated by our previous model in adult glioblastoma [14], and allows it to be adapted for personalized medicine by pairing it with patient cells isolated at the time of surgery.

In summary, the DMG‐BBTB platform provides a highly relevant resource for the scientific community studying DMG and other brain tumors. We have developed a high‐fidelity model that faithfully recapitulates the transcriptomic features of patient DMG microvasculature and features perfusable vasculature, addressing a need for a human‐representative model poised for functional testing of therapeutics. Since the vascular barrier remains intact at early stages of DMG progression, designing effective therapies that can successfully traffic to the tumor through the BBTB requires robust models like this one. As the standard‐of‐care for DMG remains radiation therapy [2], examining the vascular response to radiation is important to understand mechanisms of resistance in the DMG BBTB [56]. Future studies using the DMG‐BBTB model will examine the influence of therapeutic conditions on the tumor microenvironment, including radiation therapy and chemotherapy, as well as screening of nanotherapeutic modalities. Overall, the high spatiotemporal resolution of our DMG‐BBTB model represents a significant advance in preclinical modeling, enabling in‐depth investigation of BBTB interactions to accelerate the development of DMG therapeutics.

## 4 Conclusion

Currently, there are no preclinical in vitro models of diffuse midline glioma to study the blood–brain–tumor barrier with functional human vasculature. To address this limitation, we developed a self‐assembling microvascular model of the human DMG BBTB that recapitulates the vascular networks and signaling pathways observed in DMG tumors. We demonstrate that the DMG‐BBTB model faithfully recapitulates the 3D structure and transcriptomic profile of human brain microvasculature, benchmarked with established patient datasets. This robust system enables both quantitative solute perfusion studies and in‐depth investigation of key vascular‐tumor interactions. Our DMG‐BBTB model advances preclinical modeling of vascular‐tumor interactions for DMG, offering a human‐representative platform ideal for investigating the tumor microenvironment and developing novel therapeutics.

## 5 Materials and Methods

### 5.1 Materials

Dow Sylgard polydimethylsiloxane elastomer kits were purchased from Ellsworth Adhesives (catalog #184 SIL ELAST KIT 0.5KG). Fibrin (catalog #F8630‐1G), Thrombin (catalog #T4648‐1KU), Fibronectin (catalog #FC010‐5MG), and LiberaseTM (catalog #LIBTM‐RO) were purchased from Millipore Sigma. VascuLife medium for endothelial cells and devices was purchased from LifeLine Cell Technologies (catalog #LL‐0003). Primary human brain microvascular endothelial cells were purchased from Angioproteomie (catalog #cAP‐0002GFP). Primary human astrocytes (catalog #1800) and primary human brain vascular pericytes (catalog #1200) were purchased from ScienCell. Astrocyte medium (catalog #1801), Pericyte medium (catalog #1201), and poly‐l‐lysine (catalog #0403) were purchased from ScienCell. Tumor stem media components neurobasal‐A medium (catalog #10888‐022), D‐MEM/F‐12 (catalog #11330‐032), HEPES (catalog #15630‐080), MEM sodium pyruvate solution (catalog #11360‐070), MEM non‐essential amino acids solution (catalog #11140‐050), GlutaMAX‐I (catalog #35050‐061) and B‐27 supplement without vitamin A (catalog #12587‐010) were purchased from Invitrogen. Human recombinant TNFα (catalog #PHC3016), puromycin (catalog #J67236.8EQ), lot‐matched fetal bovine serum (catalog #A5670401), and tetramethylrhodamine‐labeled dextrans (catalog #D1842 and catalog #D1819) were also purchased from Invitrogen. hEGF (catalog #7800), hFGF (catalog #78003), heparin (catalog #07980), and Accutase (catalog #07920) were purchased from StemCell Technologies. Custom lentivirus construct encoding pRCD‐CMV‐nanoLuc‐2A‐TagRFP‐2A‐Puro was purchased from Cellecta. TrypLE (catalog #12605028), secondary antibodies (catalog #A‐21245, #A‐31553 and #A‐31556), and DAPI (catalog #D3571) were purchased from ThermoFisher Scientific. Immunofluorescence blocking buffer (catalog #12411), antibody dilution buffer (catalog #12378), anti‐PDGFRb (catalog #3169), anti‐GFAP (catalog #3670), anti‐CD44 (catalog #37259) were purchased from Cell Signalling Technologies.

### 5.2 Cell Culture

All cell culture procedures were performed using an aseptic technique in a laminar flow cell culture hood while wearing personal protective clothing. Cells were grown in an atmosphere of 5% (v/v) carbon dioxide/air in 37°C humidified incubators. Primary human brain microvascular endothelial cells (HBMEC) with a fluorescent GFP reporter were obtained from Angio‐Proteomie (catalog #cAP‐0002‐GFP, lot #2021040202) and cultured following the manufacturer's recommended protocol, including pre‐treating cultureware with Quick Coat Solution (Angio‐Proteomie, catalog #cAP‐01). Primary human astrocytes (HA) derived from the cerebral cortex (ScienCell, catalog #1800, lot #37618) and primary human brain vascular pericytes (HBVP) (ScienCell, catalog #1200, lot #34237) were cultured following the vendor's recommended protocol, including pre‐treating cultureware with 0.1% poly‐l‐lysine (ScienCell, catalog #0403). Primary cells were expanded and used between 2–5 passages from original stocks and confirmed mycoplasma negative prior to seeding.

Patient‐derived H3K27‐altered DMG brain tumor cell lines, BT245 and BT869, were obtained as a gift from the Dana–Farber Cancer Institute Center for Patient Derived Models. Cells were cultured as a neurosphere suspension and maintained in ultralow‐adherent flasks. Neurospheres were grown in neural stem cell media, comprised of 500 mL neurobasal‐A medium (Invitrogen, catalog #10888‐022), D‐MEM/F‐12 (Invitrogen, catalog #11330‐032), 500 mL of 1 M HEPES buffer solution (Invitrogen, catalog #15630‐080), 10 mL 100 mM 100X MEM sodium pyruvate solution (Invitrogen, catalog #11360‐070), 10 mL 10 mM 100X MEM non‐essential amino acids solution (Invitrogen, catalog #11140‐050), 10 mL GlutaMAX‐I supplement (Invitrogen, catalog #35050‐061), 10 mL 100X penicillin–streptavidin (Gibco, catalog #15140‐122), and 10 mL 50X B‐27 supplement without vitamin A (Invitrogen, catalog #12587‐010). Aliquots of 50 mL of the resulting base medium were supplemented with growth factors to create a working media concentration of 20 ng mL^−1^ hEGF (StemCell Technologies, catalog #7800), 20 ng mL^−1^ hFGF (StemCell Technologies, catalog #78003), and 2 µg mL^−1^ heparin (StemCell Technologies, catalog #07980) for BT245, and an additional 10 ng mL^−1^ PDGFRa (Shenandoah Biotech, catalog #100‐16) and 10 ng mL^−1^ PDGFRb (Shenandoah Biotech, catalog #100‐18) for BT869 media. Cells were mycoplasma‐negative and STR‐verified prior to device seeding.

### 5.3 Tumor RFP‐Reporter Transfection

To enable live visualization of tumor growth, the BT245 tumor line was transfected with a TagRFP fluorescent reporter using a custom lentiviral construct pRCD‐CMV‐nanoLuc‐2A‐TagRFP‐2A‐Puro at 1 × 10^8^ TU (Cellecta, catalog #CLVP‐V, Lot #240108027). Titrations to determine the minimal amount of lentivirus needed for transfection and optimal dose for puromycin selection were done prior to bulk transfection. 3 million cells were seeded per well of 6‐well adherent plate and dosed with 75 µL of lentivirus per well for spinfection. Plates were spun at 2000 rpm at 30°C for 2.5 h, then left in the incubator for 14 h to recover before challenging with 1.25 ug mL^−1^ of puromycin for 72 h. Confirming untreated control viability in puromycin of 0% after 72 h, transfected cells were sorted via FACS using the FACSAria II Flow Cytometer (BD Biosciences) and gating for high intensity RFP+ cells. Retrieved RFP+ cell stocks were then frozen and/or expanded up to 6 passages.

### 5.4 Device Fabrication

The device fabrication of 3D microfluidic chips (central channel dimensions: 3 × 10 × 0.5 mm^3^) was performed using established protocols [18]. To briefly summarize, polydimethylsiloxane elastomer base and curing agent (Dow, Sylgard 184 Kit, catalog #4019862) were mixed, desiccated, and poured into microfluidic device molds. Molds were left to solidify overnight in a 70°C incubator before peeling and cutting individual devices from the mold. With the feature side facing up, a 1 mm biopsy punch was used to puncture entry holes of the middle channel and the 4 mm biopsy punch was used to puncture holes of the flanking media channels. Devices were autoclaved in a 30 min dry cycle for sterilization, and 24 mm x 40 mm coverslip glass slides were cleaned with 100% ethanol. Glass slides and devices were placed into the Atto Plasma Cleaner (Deiner Electronics) and bonded together to achieve complete microfluidic devices. Bonded devices were placed into sealed sterile petri dishes and sterilized in a 70°C dry incubator for 72 h prior to seeding.

### 5.5 Device Seeding and Culture

Device seeding was adapted from a previous protocol [18] for the cell lots used in this study. Prior to seeding the devices, HA, HBVP, HBMEC, and BT245 cells were cultured in their respective mediums to all achieve confluency on the same day. Sterile aliquots of 6 mg mL^−1^ fibrinogen (Sigma, catalog #F8630‐1G) in PBS and 100 U mL^−1^ thrombin (Sigma, catalog #T4648‐1KU) in 0.1% w/v BSA solution were prepared and kept on ice. Working solution of thrombin was made by diluting stock 1:25 in cold Vasculife Media (Lifeline Cell Technologies, catalog #LL‐0003) supplemented with 2% FBS and ¼ of the provided heparin dose, and kept on ice. Adherent cells were dislodged with TrypLE (ThermoFisher Scientific, catalog #12605028) at room temperature (RT) for 5 min, quenched, and collected in respectively labeled conical tubes. Tumor cells were dissociated with Accutase (StemCell Technolgies, catalog #07920) at RT for 5 min, quenched, and collected in a separate conical tube. Cells were counted and measured for viability prior to resuspending each cell type in thrombin solution at volumes required to achieve desired stock solutions. For BBB devices, stock solutions were prepared such that the number of seeded cells per device was approximately 180,000 HBMECs, 30,000 HAs, and 15,000 HBVPs, mixed within 15 µL of thrombin. For BBTB devices, stock solutions were prepared in thrombin such that the amounts of seeded cells were approximately 180,000 HBMECs, 30 000 HAs, 15 000 HBVPs, and 5000, 25 000, or 50 000 DMG cells mixed within 15 µL. Aliquots of 15 µL of mixed cell suspension were made and combined with 15 µL fibrinogen, immediately seeding the full 30 µL solution into the central channel of a device. Devices were seeded one by one, placed onto sterile petri dishes, and left in humidified incubator for 15 min to allow fibrin gel to fully polymerize before filling media channels with device media. Excess HBMECs were replated and later used to apply the endothelial monolayer to the side channel. On day 2–3 after initial seeding, when vessel networks begin to form but lumens have not yet opened, an endothelial monolayer was added to flank the central cell channel, and a hydrostatic pressure differential was added. Media was aspirated from the side channels, and a 40 µL fibronectin solution consisting of 1:33 dilution of fibronectin (Sigma, catalog #FC010‐5MG) in device media (Vasculife, 2% FBS, ¼ Heparin) media was added to each channel and allowed to incubate for 1 h. HBMECs from culture were brought to single cell suspension at 1 × 10^6^ cells/mL The fibronectin solution was drained from one channel, and replaced with 40 µL of HBMEC cells. Devices were tilted 90° for 15 min to allow cells to settle on the central channel interface. This step was repeated for the other media channel. The devices were returned to a flat position, and both channels aspirated. Media ports were added to one side of the microfluidic device and filled to establish pressure‐driven differential flow from a media height difference of ∼25 mm. Ports were sterilized via autoclave prior to use. Daily, device media was replaced by filling each channel with fresh device media. On day 7, vasculature is fully formed with open lumens and ready for experiments. For TNFα‐stimulated BBB devices, devices were dosed on day 7 with 1 ng mL^−1^ or 2.5 ng mL^−1^ of human recombinant TNFα (Invitrogen, catalog #PHC3016) for 1 h prior to experiments.

### 5.6 Measurement of Vessel Dimensions and Permeability

40 µL of fluorescently‐labeled dextran with a molecular weight of 3 kDa (Invitrogen, catalog #D3329), 10 kDa (Invitrogen, catalog #D1817 and AAT Bioquest, catalog #21713), 40 kDa (Invitrogen, catalog #D1842) or 70 kDa (Invitrogen, catalog #D1819 and AAT Bioquest, catalog #21715) at 0.4 mg mL^−1^ was introduced into one channel of the microfluidic devices and allowed to perfuse the self‐assembled microvascular networks briefly before immediately adding 40 µL of dextran to the opposing channel to establish equilibrium. Devices were immediately imaged using the Leica Sp8 laser‐scanning confocal microscope at three separate ROIs, each a z‐stack of 15–30 slices of 10 µm steps taken at 0 min, 6 min, and 12 min. The acquired images were processed using the provided macro we have created, for use in ImageJ to determine each device's vessel average vessel surface area, diameter, and permeability coefficient values (File S1). The provided macro aligns the z‐stack to be centered across timepoints, removing any excess slices resulting from microscope z‐drift. Permeability is calculated as a function of vessel dimensions and variance in fluorescence intensity in the matrix and over time, as previously described [57]. Briefly, the average intensity in the vessel and fibrin matrix compartments were used to measure the permeability coefficient, *P*, as the resistance per endothelial surface area (*SA*) against dextran solute flux, which is driven by a trans‐endothelial concentration difference that can be written into terms of intensity and time as follows:

(1) $$P=\frac{1}{t}\;\frac{\left(I_{m}^{t2}-I_{m}^{t1}\right)}{\left(I_{v}^{t1}-I_{m}^{t1}\right)}\;\frac{V_{m}}{SA}$$

where *t* is the time over which transport is assessed, *I*_m,t2_ – *I*_m,t1_ represents the increase in mean fluorescence intensity, assumed linearly proportional to solute concentration, in the matrix of volume *V*_m_ between time‐points and *I*_v,1_ – *I*_m,1_ represents the difference in intensity, therefore solute concentration, between the vasculature and matrix at the start of the measurement. This is repeated for each ROI of each device of each condition. Reported values in this study are hence an average value derived from the three ROIs of a device to create an *n* = 1 device value.

### 5.7 Immunofluorescence Staining

To prepare devices for immunofluorescence staining, devices were fixed on day 7 for 60 min with 4% paraformaldehyde, washed with PBS three times for 5 min, and then permeabilized for 15 min with 0.3% Triton‐X at room temperature. Devices were washed with PBS three times. From here, each step of the protocol was done with 24 h incubations at 4C on a 3D orbital shaker. Devices were blocked with immunofluorescence blocking buffer (Cell Signaling Tech, catalog #12411), washed with PBS, given primary antibodies (1:100 dilution), washed, given secondary antibody (1:200 dilution), washed, and then imaged. Devices were stained with various primary antibodies, including PDGFRb (Cell Signaling Tech, catalog #3169), GFAP (Cell Signaling Tech, catalog #3670), and CD44 (Cell Signaling Tech, catalog #37259) with their respective secondary antibodies (ThermoFisher Scientific, catalog #A‐21245, #A‐31553 and #A‐31556) and DAPI (ThermoFisher Scientific, catalog #D3571). Antibodies were diluted in antibody dilution buffer (Cell Signaling Tech, catalog #12378). Images were obtained using the Leica Sp8 laser‐scanning confocal microscope and processed in ImageJ.

### 5.8 Cell Isolation

Prior to isolation, device viability and vessel perfusability were confirmed before cell extraction by briefly perfusing vasculature with fluorescent 70 kDa dextran (0.4 mg mL^−1^) and examined under a fluorescence microscope. For preparation of samples for isolation, microcentrifuge tubes containing 50 µL of LiberaseTM (Millipore Sigma, catalog #LIBTM‐RO) solution (20% LiberaseTM in DMEM) were prepared and kept on ice. The central channel of a device was cut to expose the gel matrix containing the cells. Ultra‐fine forceps were used to carefully extract gels, placing the gel of one device within one tube, and kept on ice. Gels were digested for 45 min, mixing gently with a wide‐bore tip every 15 min, until the gels dissolved. Tubes were then spun at 300 rcf for 5 min to pellet the isolated cells, orienting the tubes so that the pellet would form at the same location in each tube. LiberaseTM solution was carefully aspirated, and cells resuspended in 30 µL of 1% BSA in ice‐cold PBS. The process was repeated for all device samples. Cells were then promptly used for library preparation and sequencing.

### 5.9 Single‐Cell RNA Sequencing

Cells viability and quantity were measured using a Luna cell counter and prepared for 3' RNA single cell analysis using GEM‐X chemistry on a Chromium X controller (10X Genomics) targeting 20 000 cells per reaction following manufacturers recommendations. cDNA was amplified 12 cycles and cDNA and libraries were assayed using an Agilent Fragment Analyzer. Libraries were quantified by qPCR (Roche Light Cycler 480II) and sequenced on an Illumina NovaSeqX for depth (150 nt forward, 150 nt reverse).

### 5.10 Data Processing and Quality Control

Single‐cell RNA sequencing (scRNA‐seq) data were collected from six samples, each corresponding to a unique experimental condition. Raw data were obtained from 10x Genomics cellranger‐8.0.1. To standardize the preprocessing pipeline, the SoupX package was employed for ambient RNA contamination correction. Raw counts were loaded using the load10X () function, and initial clustering was performed via the Seurat pipeline to inform SoupX's contamination modeling. Counts were adjusted using autoEstCont() and adjustCounts() from SoupX. Contamination‐corrected counts were used to construct new Seurat objects with the CreateSeuratObject () function [60]. Cells were filtered based on a minimum of 200 detected features in a minimum of 3 cells. All Seurat objects underwent the following preprocessing steps: normalization (NormalizeData()), identification of highly variable genes (FindVariableFeatures()), scaling (ScaleData()), dimensionality reduction via principal component analysis (RunPCA()), UMAP projection (RunUMAP()), neighbor graph construction (FindNeighbors()), and clustering (FindClusters()) at a resolution of 0.4. Library‐size normalization to 10 000 counts and log1p transformation was conducted and highly variable genes were identified using the variance‐stabilizing method, selecting the top 2000 highly variable genes. Additionally, log2‐transformed metadata for RNA feature and count information were calculated and stored in l2.n_feature and l2.n_count, respectively. A quality control summary Excel file (combined_metrics_summary.xlsx) was created from each metrics_summary.csv of each output directory of cellranger count pipeline. These metrics were then imported, and the expected doublet rate per sample was estimated using a custom Poisson‐based model. The doublet rate function accounted for the estimated number of captured cells and the chemistry type (mu = 0.144 for 10x GEM). This model estimated the fraction of doublets among captured cells and appended the results to the QC metadata. To identify potential doublets, each Seurat object was processed using the DoubletFinder package. For each sample, the doublet detection parameter pK was estimated using paramSweep() and summarizeSweep(), cluster annotations from the Seurat object were used to estimate the homotypic doublet proportion. These metrics will inform subsequent doublet classification steps. Six samples D24‐19400 through D24‐19405 were merged using the merge function from Seurat, with add.cell.ids assigned to encode biological conditions: BBB_1, BBB_2, TNFαhigh, TNFαlow, BBTB_1, and BBTB_2. The merged Seurat object was processed using the standard Seurat workflow including Normalization (NormalizeData()), Feature Selection (FindVariableFeatures()), Scaling (ScaleData()), Dimensionality Reduction (RunPCA()) followed by RunUMAP() (based on PCA, dimensions 1–20), clustering (FindNeighbors() and FindClusters()) using the Louvain algorithm and a resolution of 0.4. An elbow plot was used to guide the selection of dimensions for UMAP and clustering. Dimensionality reduction results were visualized using DimPlot(), stratified by experimental conditions and doublet classification labels. Cluster compositions across original identities were tabulated and converted to proportions. This allowed assessment of cluster structure, condition‐specific distribution, and doublet filtering. Finally, to ensure high‐quality single‐cell data prior to downstream analysis, low‐complexity or poor‐quality cells were filtered based on gene detection efficiency. For each cell, we computed the ratio of detected genes to total UMI counts (log10GenesPerUMI = log_10_[nFeature_RNA] / log_10_[nCount_RNA]) as a measure of transcript complexity. Cells with a log10GenesPerUMI below 0.75, indicative of low RNA complexity or potential missed doublets, were excluded. This filtering step retained high‐quality single‐cell transcriptomes for reliable normalization and downstream analyses.

### 5.11 Cluster Definition and Classification

Marker genes for each cluster were identified using the FindAllMarkers() function with the following parameters: assay = “RNA”, only.pos = TRUE, logfc.threshold = 0.2, and min.pct = 0.1. To prioritize robust markers for each cluster, we retained genes with adjusted *p*‐values < 0.05, log2 fold‐change > 0.4, expression in more than 30% of cells in the cluster (pct.1> 0.3), and less than 30% of cells in other clusters (pct.2< 0.3). These filtered markers were reformatted into the input structure required by the ACT (Automated Cell Type annotation) framework by concatenating gene symbols per cluster (e.g., cluster_5:GENE1, GENE2,…) and exported to a plain text file. We further validated individual cluster markers by applying FindMarkers() to detect distinct differential gene expression profiles, which were similarly filtered and formatted for ACT. To verify marker gene expression and refine cell‐type identities, feature plots were generated for canonical genes associated with endothelial cells (e.g., PECAM1, CD34), pericytes (e.g., PDGFRB, RGS5), astrocytes (e.g., GFAP, SOX2), and tumor‐specific markers (e.g., LHFPL3, OLIG2).

We used single‐cell RNA‐seq data from Crouch et al. [25] as a reference to annotate endothelial subtypes in our dataset. To refine vascular identities, we subset the Crouch reference to endothelial lineages (arterial, capillary, tip, venous, and mitotic cells), re‐processed SCTransform, PCA (20 PCs), UMAP, and nearest‐neighbor graph (resolution = 0.3), then performed SCT anchor finding and transfer (dims = 1–20). Predicted labels were added to our endothelial subset's metadata and visualized on UMAPs (split by predicted.id) to assign endothelial subclusters based on the Crouch signatures.

### 5.12 Investigating Tumor Signature

We characterized malignant transcriptional programs by projecting curated tumor metaprograms from Liu et al., 2022, onto our tumor single‐cell dataset. Briefly, we imported the Liu reference (counts and metadata), constructed a Seurat object, and restricted it to malignant annotations (AC‐like, MES‐like, OC‐like, OPC‐like‐1/‐2/‐3, cycling S and G2M). The reference and our tumor query were independently processed with SCTransform, PCA (20 PCs), and UMAP. For cross‐dataset label transfer, we computed SCT‐based transfer anchors using the intersection of variable features and a PCA‐projection workflow (FindTransferAnchors with normalization.method = “SCT”, reduction = “pcaproject”, dims = 1–20), then predicted reference labels in the query (TransferData), retaining high‐confidence assignments (prediction score ≥ 0.6) and visualizing predictions on UMAP. To build metaprogram signatures, we ran FindAllMarkers within the Liu tumor subset and selected the top 50 genes (by avg_log2FC) per annotation to define six gene sets (AC‐like, MES‐like, OC‐like, OPC‐like‐1/‐2/‐3). These signatures were scored with AddModuleScore in both the Liu reference and our tumor query, from which we derived two axes: a stemness score (max[OPC‐like‐1/‐2/‐3] − max[AC‐like, OC‐like, MES‐like]) and a lineage score (OC‐like − AC‐like). We visualized cells in the lineage‐by‐stemness plane, summarized recurrent markers with dot plots, and, where indicated, compared single‐cell pseudobulk to bulk RNA‐seq (BT245) by log2‐normalized expression to position bulk samples in the same coordinate system. For harmonized downstream analyses, we also constructed a combined Seurat object from raw counts and aligned metadata across reference and query, normalized it, rescored metaprograms, and re‐computed lineage and stemness to ensure consistent quantification across datasets.

### 5.13 Differential Expression and Pathway Enrichment Analysis

To identify transcriptional differences between the BBTB and the BBB, we performed differential gene expression and pathway enrichment analyses across individual cell types. Within each qualifying cell type, differential gene expression analysis was performed using Seurat's FindMarkers() function with a two‐sided Wilcoxon rank‐sum test using a minimum cell percentage cutoff of ≥ 10% of cells. Genes with an adjusted p‐value (FDR) < 0.05 and an absolute log2 fold change > 0.25 were considered significant. To evaluate pathway‐level differences, we conducted Gene Set Enrichment Analysis (GSEA) Version 4.3.3 using the ranked gene lists from each cell type [61]. Genes were ranked by the signal‐to‐noise metric, calculated as the product of the sign of the log2 fold change and the negative logarithm of the p‐value. These rankings were used as input for GSEA using the fgsea R package and MSigDB g version 2023.2 gene sets, including chemical and genetic perturbations (c2cgp), Canonical pathways (c2cp), hallmark (h), and Gene Ontology biological processes (c5.bp), cellular component (c5cc) and molecular function (c5mf).

### 5.14 Cell Receptor–Ligand Analysis

To investigate cell–cell communication, we applied the LIANA framework [35] on our single‐cell RNA‐seq dataset. For this analysis, Seurat objects were subset to specific experimental conditions (BBB and BBTB) and cell identity classes (endothelial subclusters) prior to running liana_wrap. LIANA integrates predictions across multiple methods and was used here to infer ligand‐receptor interactions between annotated cell populations. Interaction scores were aggregated with liana_aggregate, and statistical significance was assessed using p‐values derived from the CellPhoneDB model. Interactions were further annotated with pathway categories using the InterCell network [58], enabling classification of ligands and receptors into functional groups. We restricted analyses to significant interactions (*p* < 0.05) and excluded self‐interactions. Chord diagrams were generated with the circlize R package [59] to visualize directional communication between Tumor, Endothelial, and Stromal compartments, with pathway‐specific ribbons overlaid to indicate the functional class of each interaction. Additionally, we summarized the most recurrent ligands and receptors by computing their frequencies across significant links and highlighting the top 10 per interaction pair on subclusters (e.g., tip, capillary, arterial, venous, cycling endothelial) in BBB versus BBTB conditions, retaining only tumor‐involving interactions to characterize condition‐specific endothelial‐tumor communication.

## Author Contributions

**Kimberly Bennett**: data curation, analysis, investigation, methodology, validation, visualization, writing – draft, review, and editing. **Yann Vanrobaeys**: visualization, analysis, data curation, resources, review, and editing. **Tessy Teboh**: investigation, review, and editing. **Zakiya Smith**: investigation, review, and editing. **Anderson Scott**: software, resources, review, and editing. **Cynthia Hajal**: methodology, review, and editing. **Paula Hammond**: supervision, funding acquisition, administration, resources, review, and editing. **Joelle Straehla**: supervision, conceptualization, funding acquisition, administration, resources, review, and editing.

## Ethics Statement

All experimental procedures were conducted under approval from the Massachusetts Institute of Technology Environmental Health & Safety Committee on Assessment of Biohazards and Embryonic Stem Cell Research Oversite (CAB/ESCRO) and Seattle Children's Research Institute Institutional Biosafety Committee (IBC) in accordance with federal, state, and local guidelines. The use of human tissue‐derived cells for this study was approved by MIT CAB/ESCRO under Biological Research Registration #868 and #584, and approved by SCRI under IBC Protocol #1374. Patient‐derived cell models were originally derived through the Center for Patient Derived Models under Dana–Farber Cancer Institute IRB Protocol #10‐417 (BT869) and #10‐043 (BT245) and transferred without patient identifying information to MIT and SCRI through Materials Transfer Agreements courtesy of Dr. Keith Ligon.

## Conflicts of Interest

J.P.S. receives in‐kind support from FUJIFILM Pharmaceuticals, U.S.A. and is an advisor for Medzown. P.T.H is a member of the Board of Alector Therapeutics and the Board of Sail Biomedicine, a Flagship company, a former member of the Scientific Advisory Board of Moderna Therapeutics, and the cofounder of LayerBio. All other authors have no disclosures to report.

## Code Availability

The code for the scRNA‐seq analyses can be accessed through GitHub (https://github.com/KochInstitute‐Bioinformatics/kbenn‐Straehla_241204Ham_scRNAseq.git). The macro used for calculating permeability is attached as a supplemental file.

## Supporting information




**Supporting File 1**: adhm71474‐sup‐0001‐SuppMat.docx.

**Supporting File 2**: adhm71474‐sup‐0002‐TableS1.xlsx.

**Supporting File 3**: adhm71474‐sup‐0003‐TableS2.xlsx.

**Supporting File 4**: adhm71474‐sup‐0004‐TableS3.xlsx.

## Data Availability

The scRNA‐seq data has been deposited in the National Center for Biotechnology Information (NCBI) Gene Expression Omnibus (GEO) under accession number GSE311451. Tables with scRNAseq and LIANA results are uploaded as supplemental files. Other supporting data files can be given upon request through contacting the corresponding authors.
