# Supplementary material for: Engineered Microvascular Model of the Blood–Brain–Tumor Barrier Reveals Endothelial Remodeling in Diffuse Midline Glioma
Source: Adv Healthc Mater. 2026 Jul 28;15(37):e71474. doi: 10.1002/adhm.71474 (PMC13650788; doi:10.1002/adhm.71474)
Supplement: Supplementary file 1 [file ADHM-15-0-s003.docx]

***Engineered microvascular model of the blood-brain-tumor barrier reveals endothelial remodeling in diffuse midline glioma***

*Kimberly R. Bennett^1,2^, Yann S. Vanrobaeys^2^, Tessy Teboh^3^, Zakiya Smith^4^, Anderson Scott^2^, Cynthia Hajal^5^, Paula T. Hammond^2,4*^, and Joelle P. Straehla^2,6,7,*^*

^1^ Harvard-MIT Division of Health Sciences & Technology, Massachusetts Institute of Technology, Cambridge, MA

^2^ Koch Institute for Integrative Cancer Research, Massachusetts Institute of Technology, Cambridge, MA

^3^ Department of Biochemistry, Wellesley College, Wellesley, MA

^4^ Department of Chemical Engineering, Massachusetts Institute of Technology, Cambridge, MA

^5^ Department of Mechanical Engineering, Northeastern University, Boston, MA

^6^ Ben Towne Center for Childhood Cancer & Blood Disorders Research, Seattle Children’s Research Institute, Seattle, WA

^7^Department of Pediatrics, University of Washington, Seattle, WA

**Correspondence to Paula T. Hammond (*[hammond@mit.edu](mailto:hammond@mit.edu)*) and Joelle P. Straehla (*[Joelle.Straehla@seattlechildrens.org](mailto:Joelle.Straehla@seattlechildrens.org)*)*

**SUPPLEMENTAL DATA:**

**Figure S1: Characterization of an alternate DMG-BBTB model seeded with BT869 tumor line.** (A) Comparison between BBB, BBTB (BT245) and BBTB (BT869) devices. BBTB devices were seeded at low densities (5,000 tumor cells/device) and vessel diameters in the lateral and transverse dimensions were calculated. Values reported are averages from across 3 ROIs per device per n=1, n=4 devices per condition. Center values depict average, with error bars denoting standard deviation. Significance measured through ordinary two-way ANOVA with Šídák’s multiple comparisons test, * = p < 0.05, ns = not significant. (B) Permeability (cm s^-1^) of 70 kDa TRITC-labeled dextran in BT869 seeded devices at low and high tumor densities, mirroring BT245 studies as described in main text. Values reported are averages from across 3 ROIs per device per n=1, n=4 devices per condition. Center values depict average, with error bars denoting standard deviation. Significance measured through ordinary one-way ANOVA with Tukey’s multiple comparisons test, * = p < 0.05, ns = not significant.

**Figure S2: Engineering limit of cell density within microfluidic devices.** (A) Confocal images of vasculature within BBB and BBTB devices across tumor densities (Low – 5,000 cells, High – 25,000, Ultra High – 50,000 cells/device). Scale = 200 µm. (B) Permeability (cm s^-1^) of 70 kDa TRITC-labeled dextran in devices across tumor densities. As most devices in the ultra high condition were not fully perfusable, permeability values could not be collected for all replicates. All values reported are averages from across 3 ROIs per device per n=1, n=4 devices per condition (with the exception of ultra high condition which only had 2 semi-viable replicates i.e. less than 3 usable ROIs per device due to limited perfusion). Center values depict average, with error bars denoting standard deviation. Significance measured through ordinary one-way ANOVA with Tukey’s multiple comparisons test , * = p < 0.05.

**Figure S3: scRNAseq sample UMAP and quality controls across conditions.** (A) Unbiased clustering of pooled scRNAseq sample data, with each cluster annotated as shown in main text. (B) Mitochondrial percentage across cluster numbers across all samples. (C) Ribosomal percentage across cluster numbers. (D) RNA counts across cluster numbers. (E) RNA features across cluster numbers across all samples. (F) RNA counts by sample device origin. (E) RNA features by sample device origin.

**Figure S4: ATP-binding cassette (ABC) transporters in endothelial cells in BBB vs BBTB conditions**. Overall endothelial expression changes between BBB and BBTB are shown under “Broad”, and a breakdown of expression changes across each endothelial cluster (Venous, Cycling, Tip, Capillary, Arterial) are shown under “Subtypes”. Circle size denotes the percentage of marker expression in the group. Color indicated scaled average expression (z-score normalized per gene), where positive values reflect above-average expression and negative values reflect below-average expression relative to all cell identities shown.

**Figure S5: Solute carrier (SLC) transporters in endothelial cells in BBB vs BBTB conditions.** Overall endothelial expression changes between BBB and BBTB are shown under “Broad”, and a breakdown of expression changes across each endothelial cluster (Venous, Cycling, Tip, Capillary, Arterial) are shown under “Subtypes”. Circle size denotes the percentage of marker expression in the group. Color indicated scaled average expression (z-score normalized per gene), where positive values reflect above-average expression and negative values reflect below-average expression relative to all cell identities shown.

**Figure S6: Receptor-mediated transcytosis (RMT) transporters in endothelial cells in BBB vs BBTB conditions.** Overall endothelial expression changes between BBB and BBTB are shown under “Broad”, and a breakdown of expression changes across each endothelial cluster (Venous, Cycling, Tip, Capillary, Arterial) are shown under “Subtypes”. Circle size denotes the percentage of marker expression in the group. Color indicated scaled average expression (z-score normalized per gene), where positive values reflect above-average expression and negative values reflect below-average expression relative to all cell identities shown.

**Figure S7: Comparative analysis of Glioblastoma-associated endothelial marker expression in DMG-BBTB.** (A) Proportion of endothelial cell cluster composition between BBB and BBTB conditions. (B) UMAP feature plots of GBM-associated endothelial markers within the scRNAseq dataset. Color represents average expression of genes. (C) Overall endothelial expression changes between BBB and BBTB are shown under “Broad”, and a breakdown of expression changes across each endothelial cluster (Venous, Cycling, Tip, Capillary, Arterial) are shown under “Subtypes”. Circle size denotes the percentage of marker expression in the group. Color indicates scaled average expression (z-score normalized per gene), where positive values reflect above-average expression and negative values reflect below-average expression relative to all cell identities shown.

***Supplemental Table 1: DEGs across all cell types between BBB vs BBTB.*** An excel file that contains the full list of all differentially expressed genes between BBB and BBTB device conditions, categorized by each annotated cell population.

***Supplemental Table 2: GSEA results across all cell types between BBB vs BBTB.*** An excel file that contains the full list of all GSEA results across various referenced databases between BBB and BBTB device conditions, categorized by each annotated cell population.

***Supplemental Table 3: LIANA ligand-receptor analysis between all cell subtypes.*** An excel file that contains the full list of all ligand-receptor interactions between all cell populations.

***Supplemental File 1: Permeability calculation macro for ImageJ.*** A macro for ImageJ used to calculate permeability from confocal images of z-stacks taken at 3 positions within a device. Code is written in R and optimized for the Leica instrument file format (.LIF).
